# The population-level impact of *Enterococcus faecalis* genetics on intestinal colonization and extraintestinal infection

**DOI:** 10.1128/spectrum.00201-23

**Published:** 2023-10-09

**Authors:** Chrispin Chaguza, Anna K. Pöntinen, Janetta Top, Sergio Arredondo-Alonso, Ana R. Freitas, Carla Novais, Carmen Torres, Stephen D. Bentley, Luisa Peixe, Teresa M. Coque, Rob J. L. Willems, Jukka Corander

**Affiliations:** 1 Department of Epidemiology of Microbial Diseases, Yale School of Public Health, Yale University, New Haven, Connecticut, USA; 2 Parasites and Microbes Programme, Wellcome Sanger Institute, Wellcome Genome Campus, Cambridge, United Kingdom; 3 Department of Biostatistics, Faculty of Medicine, University of Oslo, Oslo, Norway; 4 Norwegian National Advisory Unit on Detection of Antimicrobial Resistance, Department of Microbiology and Infection Control, University Hospital of North Norway, Tromsø, Norway; 5 Department of Medical Microbiology, University Medical Center Utrecht, Utrecht, the Netherlands; 6 UCIBIO-Applied Molecular Biosciences Unit, Laboratory of Microbiology, Department of Biological Sciences, REQUIMTE Faculty of Pharmacy, University of Porto, Porto, Portugal; 7 Associate Laboratory i4HB, Institute for Health and Bioeconomy, Faculty of Pharmacy, University of Porto, Porto, Portugal; 8 TOXRUN, Toxicology Research Unit, University Institute of Health Sciences, CESPU, CRL, Gandra, Portugal; 9 Department of Food and Agriculture, Area of Biochemistry and Molecular Biology, University of La Rioja, Logroño, Spain; 10 Department of Microbiology, Ramón y Cajal University Hospital, Ramón y Cajal Institute for Health Research (IRYCIS), Madrid, Spain; 11 CIBER in Infectious Diseases (CIBERINFEC), Madrid, Spain; 12 Department of Mathematics and Statistics, Helsinki Institute of Information Technology, University of Helsinki, Helsinki, Finland; University of Valencia, Paterna, Valencia, Spain

**Keywords:** microbial genomics, infectious disease, genome-wide association study, bacteria

## Abstract

*Enterococcus faecalis* is a commensal bacterium of the human gastrointestinal tract that causes opportunistic infections. The *E. faecalis* genetic changes associated with pathogenicity, particularly gut-to-bloodstream translocation, remain poorly understood. Here, we performed a genome-wide association study (GWAS) of 736 whole-genome sequences of fecal and bloodstream *E. faecalis* isolates from hospitalized and nonhospitalized individuals, respectively, to identify *E. faecalis* genetic signatures associated with the patient’s hospitalization status and body isolation source. We found that infection by hospitalization status and extraintestinal infection are heritable traits, with ~40% and ~30% of their variation explained by *E. faecalis* genetics, respectively. Furthermore, a GWAS using linear mixed models did not pinpoint any clear overrepresentation of individual genetic changes by hospitalization status or body isolation source after controlling for the population structure. However, we observed elevated signals in a genomic region containing a prophage element. However, the lineages themselves and their associated virulence factors and antibiotic resistance genes showed variable frequency among blood and fecal isolates and in hospitalized and nonhospitalized individuals. Altogether, our findings indicate that *E. faecalis* infection by hospitalization status and body sites is partially influenced by the overall genetic background of the isolates and antibiotic resistance patterns rather than genetic variation at individual loci, which suggests a greater role of other host and environmental factors and ultimately the opportunistic pathogenic lifestyle of this versatile host generalist bacterium.

**IMPORTANCE:**

*Enterococcus faecalis* causes life-threatening invasive hospital- and community-associated infections that are usually associated with multidrug resistance globally. Although *E. faecalis* infections cause opportunistic infections typically associated with antibiotic use, immunocompromised immune status, and other factors, they also possess an arsenal of virulence factors crucial for their pathogenicity. Despite this, the relative contribution of these virulence factors and other genetic changes to the pathogenicity of *E. faecalis* strains remain poorly understood. Here, we investigated whether specific genomic changes in the genome of *E. faecalis* isolates influence its pathogenicity—infection of hospitalized and nonhospitalized individuals and the propensity to cause extraintestinal infection and intestinal colonization. Our findings indicate that *E. faecalis* genetics partially influence the infection of hospitalized and nonhospitalized individuals and the propensity to cause extraintestinal infection, possibly due to gut-to-bloodstream translocation, highlighting the potential substantial role of host and environmental factors, including gut microbiota, on the opportunistic pathogenic lifestyle of this bacterium.

## INTRODUCTION

*Enterococcus faecalis* is a versatile generalist commensal bacterium that colonizes the gastrointestinal tract and other niches in humans and animals and survives in the environment, including nosocomial settings (1). *E. faecalis* is a subdominant core member of the human gut microbiota, usually acquired early after birth, and its origin dates to the Paleozoic era ~400 to 500 million years ago (2). Although *E. faecalis* predominantly exhibits a commensal lifestyle, it is a conditional or opportunistic pathogen (3, 4). It causes life-threatening opportunistic infections, including bacteremia, endocarditis, intra-abdominal infection, pneumonia, and meningitis infections typically associated with high mortality (5, 6). Since the 1970s, *E. faecalis* has emerged as a leading cause of community-acquired and nosocomial infections, most of which have become increasingly difficult to treat due to intrinsic and acquired antibiotic resistance, making it a major threat to public health globally (4, 6 – 9). Such increasing antibiotic resistance has reignited calls to develop enterococcal vaccines.

The commensal-to-pathogenic switch of *E. faecalis* is marked by its overgrowth in the gut and subsequently translocation into the bloodstream via the intestinal epithelium (10). Such extraintestinal translocation can lead to bacteremia, infective endocarditis, and infections in other distal tissues from the intestines. However, the specific mechanisms driving *E. faecalis* bloodstream invasion, survival, and virulence are still being uncovered (3, 5, 11, 12). Observational studies have shown that antibiotics, such as cephalosporins, promote overgrowth and extraintestinal translocation of *E. faecalis* into the bloodstream (13, 14), an observation supported by *in vivo* murine experimental models (14 – 16). Such overgrowth of *E. faecalis* reflects the impact of ecological side effects of broad-spectrum antibiotics in driving dysbiosis of the gut microbiota*,* a phenomenon similarly observed with *Clostridioides difficile* (formerly known as *Clostridium difficile*) (17, 18). *E. faecalis* also harbors a diverse arsenal of putative virulence factors (19 – 21), which foster its adaptation and survival in the harsh clinical and midgut environments and potentially promote extraintestinal translocation into the bloodstream. These virulence factors appear to be enriched in the dominant epidemic *E. faecalis* lineages (22, 23), highlighting their importance to the success of these clones. For example, the gelatinase (*gelE*) gene encodes a metalloprotease exoenzyme commonly associated with epidemic clones (22) and is important for infective endocarditis (24) and extraintestinal translocation into the bloodstream (25). Other exotoxins, namely, hemolysin and enterococcus surface protein, are also important for virulence in endocarditis (26) and biofilm formation (27), respectively, although the role of the former on intestinal colonization and translocation has been questioned (28, 29 ). Acquisition of extrachromosomal elements, including pathogenicity islands (30, 31) and plasmids (32), has also been associated with virulence and survival in nosocomial settings (33). Understanding the distribution of these known and novel *E. faecalis* virulence factors in strains sampled from different tissues and individuals with contrasting pathogenicity could potentially reveal mechanisms for enterococcal pathogenicity and uncover therapeutic targets.

Remarkable advances in whole-genome sequencing and computational biology have revolutionized population genomics since the sequencing of the first enterococcal genome (34). To date, the feasibility of large-scale whole-genome sequencing and analysis has facilitated detailed population-level studies to uncover the genetic basis of bacterial phenotypes (35). For example, the application of genome-wide association studies (GWAS) to bacteria has revealed genetic variants associated with diverse phenotypes, including antimicrobial resistance (36), host adaptation (37), and pathogenicity (38). A key feature of the GWAS approach is that it can identify novel genetic variants associated with phenotypes through systematic genome-wide screening, which does not bias the analysis toward “favorite” genes and mutations commonly studied in different laboratories. Although previous studies have attempted to compare the genetic and phenotypic differences between *E. faecalis* isolates causing intestinal colonization and invasive disease (39), clinical and nonclinical strains (40), and isolates of diverse origins (41), these studies were limited by the small sample sizes and use of low-resolution molecular typing methods such as pulsed-field gel electrophoresis. Recent studies of *E. faecalis* and *Enterococcus faecium* species identified unique mutations associated with outbreak strains, highlighting the potential effects of specific genetic changes on pathogenicity (12, 42). Despite the increasing affordability of population-scale microbial sequencing, the genetic basis of *E. faecalis* infection in individuals with different hospitalization statuses, i.e., pathogenicity and extraintestinal infection, including those due to extraintestinal translocation, remains poorly understood. The application of GWAS approaches to discover the genetic changes driving the pathogenicity and virulence of *E. faecalis* could expedite antibiotic and vaccine development.

Here, we leveraged a collection of 736 whole-genome sequenced *E. faecalis* isolates sampled from the feces and blood specimens of hospitalized and nonhospitalized individuals (43). We undertook a GWAS of the isolates to investigate if specific genomic variations, including single-nucleotide polymorphisms (SNPs) and insertions/deletions, were associated with infection by hospitalization status and body isolation source. We show a predominantly higher differential abundance of virulence factors and antibiotic resistance in *E. faecalis* isolates from hospitalized than from nonhospitalized individuals, as well as isolates from blood than from feces. This largely reflects the effects of the genetic background or lineages, as no specific individual genetic changes showed population-wide effects on the infection of individuals by hospitalization status or isolation source. Additionally, we found that infection in individuals depends on their hospitalization status and extraintestinal infection, which are heritable traits partially explained by *E. faecalis* genetics. Altogether, our findings provide evidence suggesting that the collective effects of several genetic variants, genetic background or lineages, and gut ecological factors drive the pathogenicity and extraintestinal infection of *E. faecalis* rather than the population-wide effects of individual bacterial genetic changes. These findings have broader implications for *E. faecalis* disease prevention strategies, specifically the need to target all genetic backgrounds when designing vaccines to achieve optimal protection against severe enterococcal invasive diseases.

## RESULTS

### Clinical and genomic characteristics of *E. faecalis* isolates

**Fig 1:**
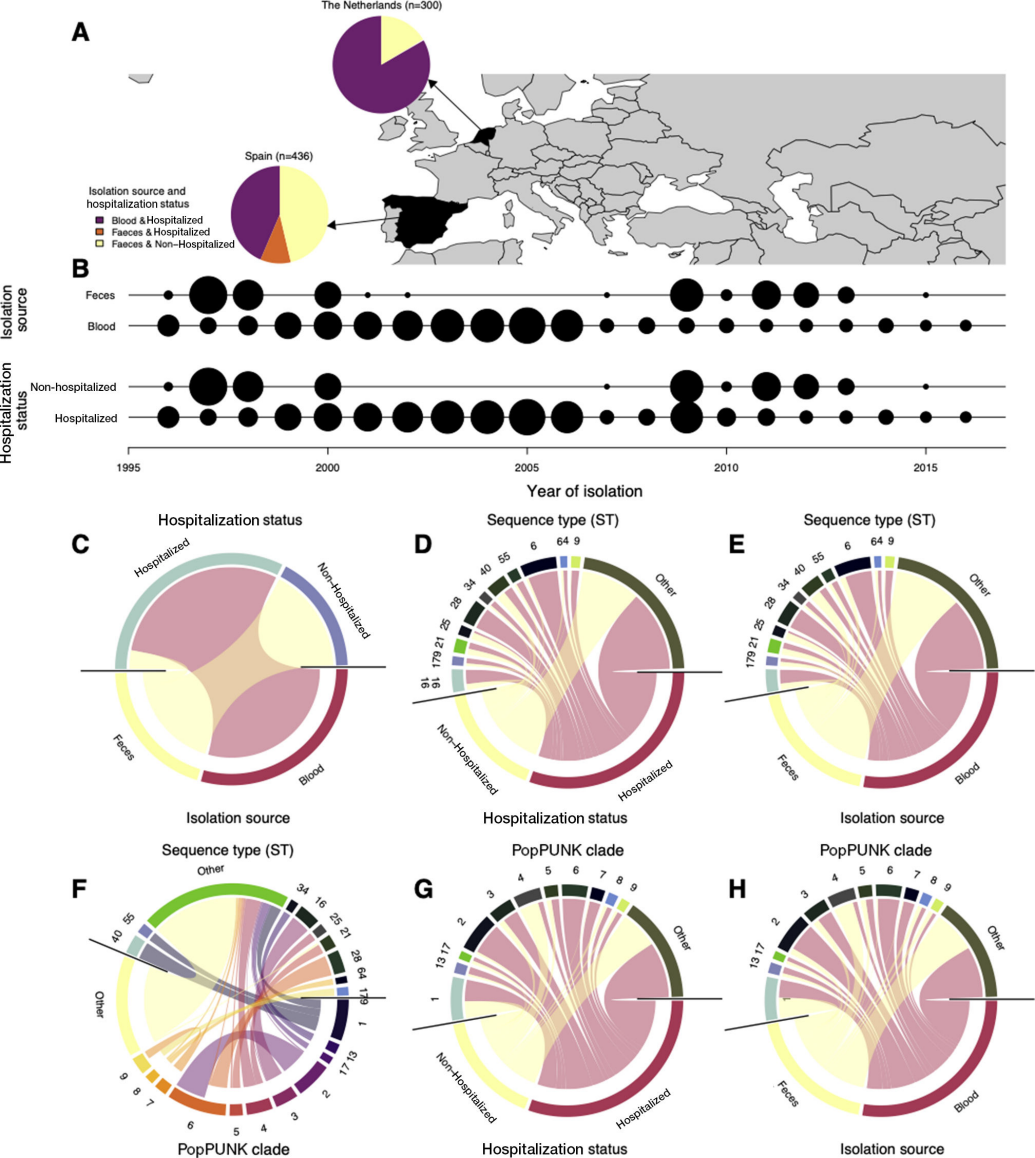
Characteristics of *E. faecalis* isolates included in this study. (**A**) Summary of the convenient sample of *E. faecalis* isolates collected from individuals in the Netherlands and Spain, showing the frequency of isolates from hospitalized and nonhospitalized individuals, and blood and feces. The map was generated using the R package rworldmap. (**B**) Temporal distribution of the *E. faecalis* isolates in each country. The radius of each black circle represents the square root of the number of isolates selected per year for whole-genome sequencing. (**C**) Association between *E. faecalis* isolates by the sampling site and pathogenicity or hospitalization status. (**D**) Association of the *E. faecalis* isolates by hospitalization status and sequence type (ST) based on the multi-locus sequence typing scheme approach. (**E**) Association of the *E. faecalis* isolates by ST and isolation source. (**F**) Association of the *E. faecalis* isolates by STs and clades or lineages defined using Population Partitioning Using Nucleotide K-mers (PopPUNK) by Pöntinen et al. (43). (**G**) Association of the *E. faecalis* isolates by hospitalization status and PopPUNK clades or lineages. (**H**) Association of the *E. faecalis* isolates by body isolation source and the PopPUNK clades or lineages.

To investigate the population genomics of *E. faecalis* pathogenicity, marked by infection of individuals by hospitalization status and body isolation source*,* we compiled a data set of 736 whole genome sequences of *E. faecalis* isolates sampled from blood and fecal specimens of hospitalized and nonhospitalized individuals between 1996 and 2016 (43) (Fig. 1A; Data Set S1). We included isolates from countries where both fecal and bloodstream isolates were collected, but not necessarily from the same individual. In total, our final data set comprised isolates from Europe: the Netherlands (*n* = 300) and Spain (*n* = 436) (Fig. 1B). By infection of individuals, 485 isolates were obtained from hospitalized patients, while 251 isolates were sampled from nonhospitalized individuals (Data Set S1). Regarding the isolation of *E. faecalis* from human body sites, 440 isolates were sampled from the blood, while 296 isolates were from feces. Nearly all the isolates from nonhospitalized individuals were collected from feces, while those from blood were from hospitalized individuals. Such a discrepancy in sampling of the *E. faecalis* isolates by hospitalization status and body isolation source reflected the fact that invasive sampling, such as collecting blood samples, was less likely to be performed for the nonhospitalized than the hospitalized patients. In addition, considering that *E. faecalis* is a major cause of nosocomial infections, there is a greater likelihood that the isolation of *E. faecalis* in hospitalized individuals may be a consequence of acquisition in the hospital environment by already hospitalized individuals with weaker immunity rather than only a reflection of its intrinsic pathogenicity (Fig. 1C).

### *E. faecalis* infections by hospitalization status and body isolation source are heritable phenotypes predominantly explained by genetic background or lineages

To assess the overall genetic basis of the infection in individuals with different hospitalization statuses, we quantified the proportion of the variability in the phenotypes explained by *E. faecalis* genetics. We calculated the narrow-sense heritability (*h* ^2^) based on the kinship matrix generated using unitig sequences (44). After adjusting for the geographical origin and year of isolation of the isolates, we found a heritability of *h* ^2^ = 0.40 [95% confidence interval (CI): 0.23 to 0.57] and *h* ^2^ = 0.30 (95% CI: 0.15 to 0.45) for infection by hospitalization status and body isolation source, respectively. Next, we calculated the heritability for infection of individuals by hospitalization status and body isolation source using only the Spanish cohort, which had an even number of isolates from hospitalized and nonhospitalized individuals as well as from blood and feces. We found consistent, but slightly higher, estimates of heritability for both infection of individuals by hospitalization status (*h* ^2^ = 0.43, 95% CI: 0.23 to 0.63) and body isolation source (*h* ^2^ = 0.28, 95% CI: 0.12 to 0.45) than estimated based on the combined data set. These findings suggest that *E. faecalis* infections by hospitalization status and body isolation source are moderately heritable traits partially explained by genetics.

### Infections of individuals with *E. faecalis* by hospitalization status and body isolation source vary across lineages

**Fig 2:**
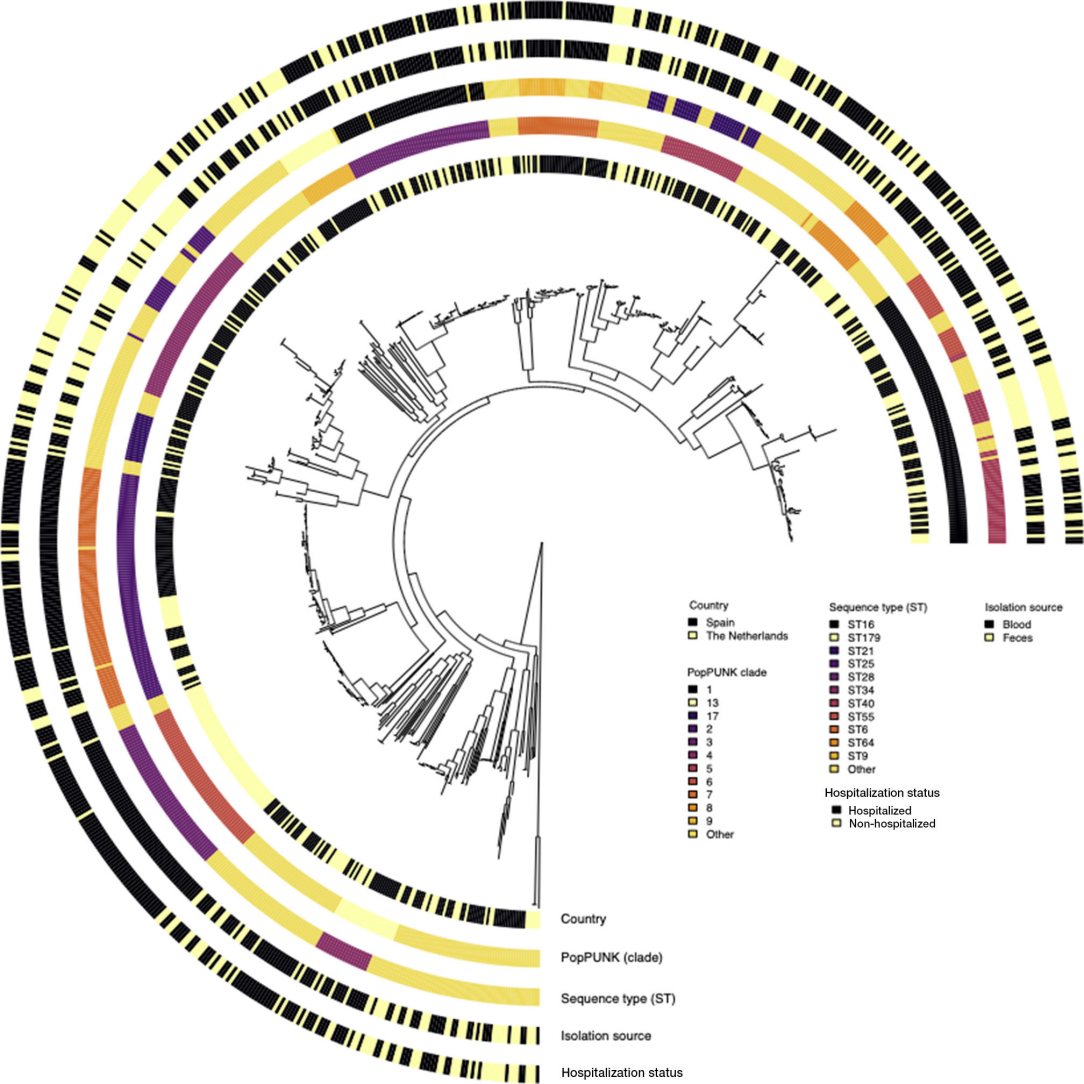
Maximum-likelihood phylogenetic tree of 736 *E. faecalis* isolates from the Netherlands and Spain. Each circular ring at the tip of the phylogenetic tree, from innermost to outermost, represents the country of origin for each *E. faecalis* isolate (the Netherlands and Spain), clade or lineage defined by the PopPUNK genomic sequence clustering framework (47) by Pöntinen et al. (43), ST based on the *E. faecalis* MLST scheme (48), body isolation source (blood and feces), and pathogenicity defined based on hospitalization status (hospitalized and nonhospitalized). The phylogeny was rooted at the midpoint of the longest branch between the two most divergent *E. faecalis* isolates.

We sought to investigate the distribution of the hospitalization and body isolation source phenotypes in the context of the *E. faecalis* population structure. We generated a maximum-likelihood phylogenetic tree using 251,983 core genome SNPs, exclusively containing nonambiguous nucleotide and deletion characters, and annotated it with the hospitalization status and body isolation source phenotypes. The isolates were widely distributed across different genetic backgrounds based on the country of origin as well as body isolation source and hospitalization status, a finding consistent with the literature that the severity of *E. faecalis* infections is not restricted to specific lineages, in contrast to the genetic separation between commensal and hospital-adapted lineages observed in *E. faecium* (45, 46) (Fig. 2). We then performed an in-depth analysis of the *E. faecalis* population structure using lineage definitions based on the Population Partitioning Using Nucleotide K-mers (PopPUNK) genomic sequence clustering framework (47) by Pöntinen et al. (43). Our isolates clustered into 96 clades, which corresponded to 121 sequence types (STs) or clones defined by the *E. faecalis* multi-locus sequence typing scheme (MLST) (48) (Fig. 2). There was no single dominant ST present at a frequency of >50% compared to the others among the isolates sampled from hospitalized and nonhospitalized patients and isolates from feces and blood (*P* > 0.05) (Fig. 1D and E). As expected, the clusters defined by the MLST scheme were concordant with the PopPUNK clades or lineages, although the latter were less granular than the former as they are defined based on genome-wide variation and therefore are robust to subtle genomic variation (Fig. 1F). Therefore, as similarly observed with the STs, there was no single dominant clade present at a frequency of >50% compared to the rest associated with the *E. faecalis* isolates from hospitalized and nonhospitalized patients and isolates from feces and blood (*P* > 0.05) (Fig. 1G and H).

**Fig 3:**
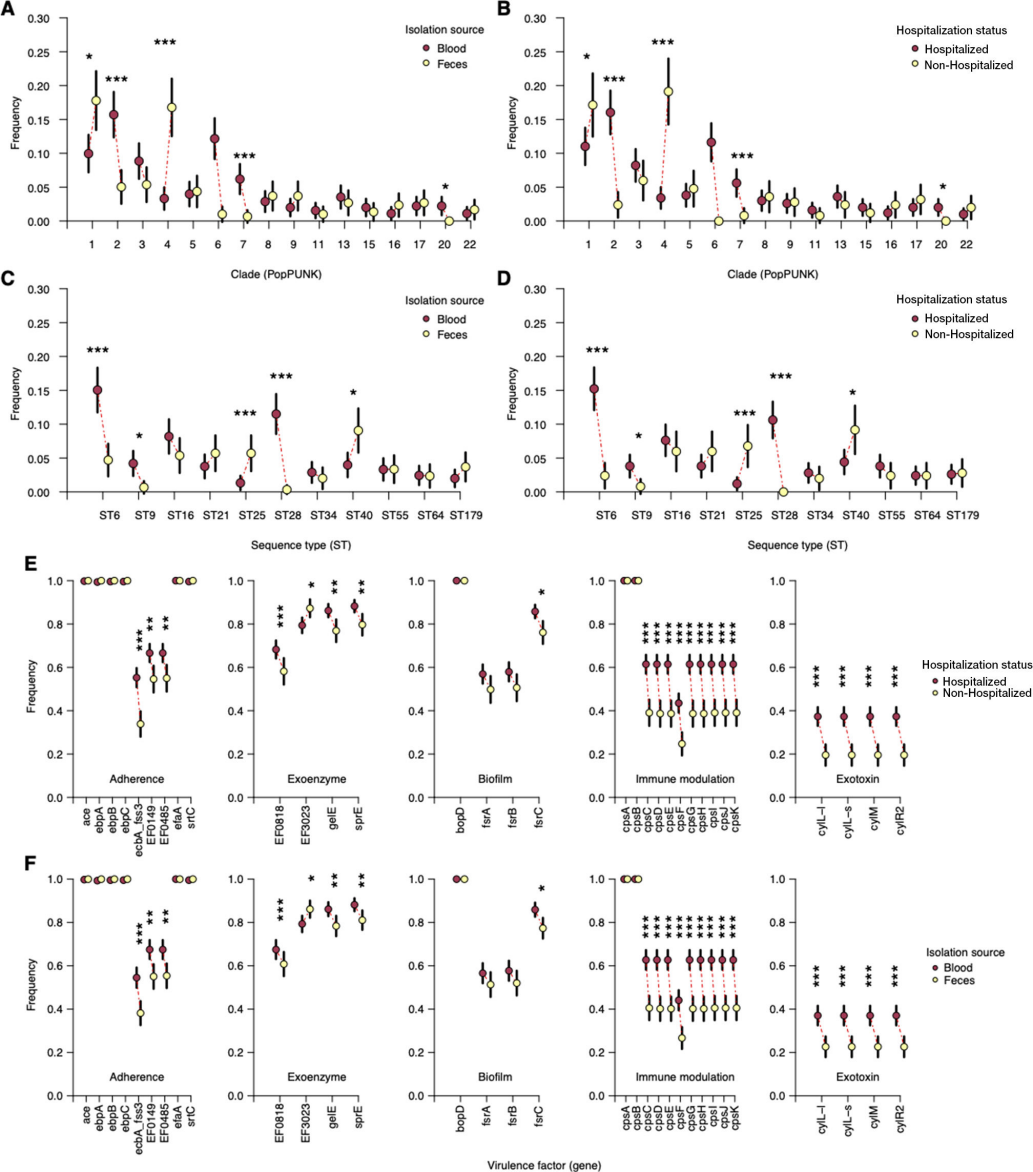
Relative abundance of *E. faecalis* lineages and virulence factors by hospitalization status and body isolation source. (**A**) Relative frequency of *E. faecalis* clades or lineages among isolates collected from blood and feces. (**B**) Relative frequency of *E. faecalis* clades or lineages among isolates collected from hospitalized and nonhospitalized individuals. (**C**) Relative frequency of *E. faecalis* ST among isolates collected from blood and feces. (**D**) Relative frequency of *E. faecalis* STs among isolates collected from hospitalized and nonhospitalized individuals. (**E**) Relative frequency of a catalog of known *E. faecalis* virulence factors from the virulence factor database (VFDB) (49) among hospitalized and nonhospitalized individuals. (**F**) Relative frequency of *E. faecalis* virulence factors from VFDB among isolates collected from blood and feces. All the error bars in each plot represent 95% binomial proportional confidence intervals. The asterisks above the frequency of some genes show the statistical significance of the difference in proportions based on the test for the equality of two proportions defined as follows: *P* < 0.001 (***), *P* < 0.01 (**), and *P* < 0.05 (*).

We then compared the relative frequency of individual STs and PopPUNK clades between isolates collected from hospitalized patients and nonhospitalized individuals. We found three clades more common in hospitalized patients than in nonhospitalized individuals, namely, clades 2 (adjusted *P* = 1.20 × 10^−05^), 6 (adjusted *P* = 4.80 × 10^−08^), and 7 (adjusted *P* = 0.0003). In contrast, two clades, clade 1 (adjusted *P* = 0.027) and clade 4 (adjusted *P* = 3.40 × 10^−10^), were more common in nonhospitalized individuals than in hospitalized patients (Fig. 3A; Table S1). Due to the correlation between the hospitalization status of the individuals and the isolation source, we found similar patterns in the relative abundance of the clades between blood and fecal isolates (Fig. 3B; Table S2). We found a higher abundance of ST6 (clade 2; adjusted *P* = 1.50 × 10^−05^), ST9 (clade 7; adjusted *P* = 0.0082), and ST28 (clade 6; adjusted *P* = 1.20 × 10^−08^) among hospitalized patients than among nonhospitalized individuals (Fig. 3C and D; Table S2). Similar patterns were observed among isolates sampled from blood compared to feces. Conversely, we found that ST25 (clade 4) was enriched in nonhospitalized patients compared to nonhospitalized individuals (adjusted *P* = 0.014) as well as in isolates sampled from blood compared to feces (adjusted *P* = 7.80 × 10^−05^) (Fig. 3C and D; Table S2). Together, these findings suggest that certain *E. faecalis* genetic backgrounds are overrepresented in patients by hospitalization status and isolation source, suggesting that the lineage, which correlates with the presence of virulence factors and antibiotic resistance determinants, partially influences extraintestinal infection of *E. faecalis*.

### Only a few virulence factors show variable prevalence in individuals with different hospitalization statuses and isolation sources

As a generalist host species, *E. faecalis* exhibits high levels of recombination (48), which may facilitate the acquisition of genes promoting colonization and virulence, driving the success of its clones (23) We hypothesized that certain known virulence factors would be enriched among *E. faecalis* isolates from hospitalized patients, especially those with bloodstream infection, compared to nonhospitalized individuals without bloodstream infection. We used a candidate gene approach to compare the enrichment of a catalog of *E. faecalis* virulence factors obtained from the virulence factor database (VFDB) (49) by individuals’ hospitalization status and body isolation source. We found that three genes, namely, ecbpA (adjusted *P* = 5.70 × 10^−08^), EF0149 (adjusted *P* = 0.0019), and EF0485 (adjusted *P* = 0.0026), which play a role in epithelial surface adherence, were more common in extraintestinal infection than in intestinal colonization (Fig. 3E and F; Table S3). No genes encoding known exoenzyme and biofilm-associated proteins showed differential enrichment in either hospitalized patients relative to nonhospitalized individuals or extraintestinal infection compared to intestinal colonization (Fig. 3E and F; Table S3). However, all four exotoxin-encoding genes were enriched in hospitalized compared to nonhospitalized individuals, namely, *cylL-l* (adjusted *P* = 1.20 × 10^−06^), *cylL-s* (adjusted *P* = 1.20 × 10^−06^), *cylM* (adjusted *P* = 1.20 × 10^−06^), and *cylR2* (adjusted *P* = 1.20 × 10^−06^) (Fig. 3E; Table S3). Similar patterns were observed among the isolates sampled from extraintestinal infection and intestinal colonization (Fig. 3F; Table S3). Additionally, nine capsule biosynthesis genes (*cpsC* to *cpsK*) were more common among hospitalized than among nonhospitalized individuals as well as isolates from the extraintestinal infection than from the intestinal colonization (Fig. 3E and F; Table S3). These findings are partly consistent with previous studies (22, 23), although the present study investigated a larger catalog of virulence factors. Therefore, we conclude that certain virulence factors are associated with individuals with different hospitalization statuses and possibly promote extraintestinal translocation of *E. faecalis* into the bloodstream in hospitalized individuals.

### Distribution of antibiotic resistance genes in *E. faecalis* isolates by hospitalization status and body isolation source

**Fig 4:**
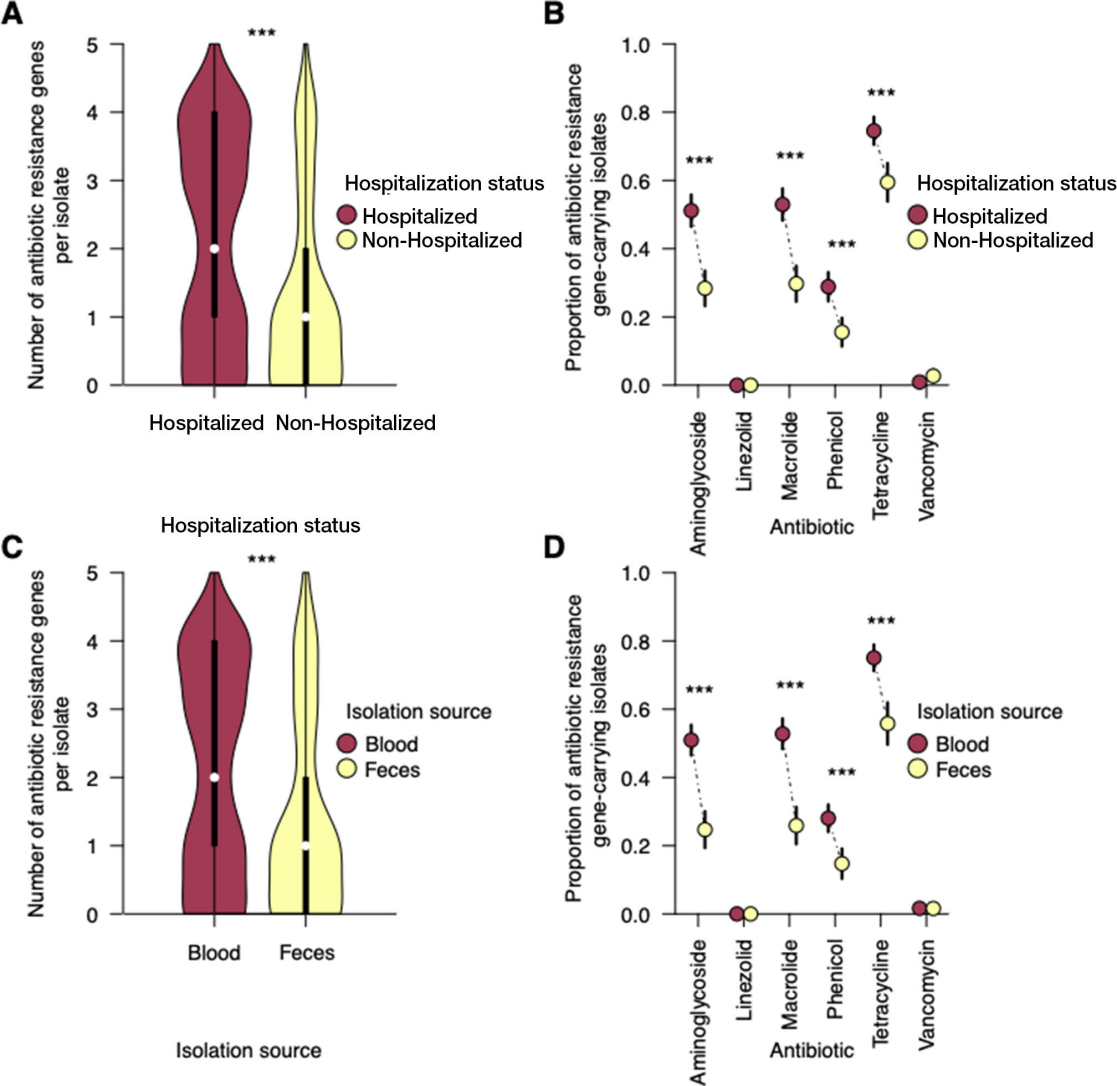
The abundance of *E. faecalis* antibiotic resistance genes by hospitalization status and body isolation source. (**A**) Distribution of the number of antibiotic resistance genes (see Materials and Methods) per *E. faecalis* isolates from hospitalized and nonhospitalized individuals. (**B**) Relative abundance or frequency of genotypically resistant *E. faecalis* isolates from hospitalized and nonhospitalized individuals. (**C**) Distribution of the number of antibiotic resistance genes per *E. faecalis* isolates collected from blood and feces. (**D**) Relative abundance or frequency of genotypically resistant *E. faecalis* isolates collected from blood and feces. All the error bars in each plot represent 95% binomial proportional confidence intervals.

Hospitalized patients are more exposed to antibiotics in hospitals than nonhospitalized individuals, as more antibiotics are used in hospital settings than outside. Therefore, it is likely that *E. faecalis* isolates from hospitalized patients are more likely to have acquired resistance than isolates from nonhospitalized individuals. Because most patients were probably hospitalized because of other complaints and developed the *E. faecalis* infection during hospitalization, we hypothesized that *E. faecalis* isolates sampled from hospitalized individuals and extraintestinal infection would show a higher frequency of antibiotic resistance traits than isolates from nonhospitalized individuals and intestinal colonization. The rationale behind this hypothesis was that antibiotic-susceptible *E. faecalis* strains are more likely to be cleared from the gut following antibiotic use, leaving more space for the surviving antibiotic-resistant strains to cause extraintestinal infection and subsequently cause severe disease (Fig. 1C; Table S4). This would be due to the surviving antibiotic-resistant strains. We investigated this hypothesis by comparing the abundance of antibiotic resistance genes for seven antibiotic classes, namely, glycopeptides (vancomycin), aminoglycosides, macrolides, tetracyclines, phenicols, and oxazolidinones (linezolid), in *E. faecalis* isolates from hospitalized and nonhospitalized individuals, blood, and feces. Regressing the number of antibiotic classes susceptible to the hospitalization status while adjusting for the country of origin showed resistance to more antibiotic classes among isolates from hospitalized than from nonhospitalized individuals (effect size *β* = 1.43, *P* < 3.63 × 10^−12^) (Fig. 4A; Table S4). As expected, due to the correlation between hospitalization status and body isolation source (Fig. 1C; Table S4), we found a similar pattern for isolation source, i.e., isolates from blood harboring resistance traits to a higher number of antibiotic classes than isolates from feces (effect size *β* = 1.37, *P* = 2.89 × 10^−10^) (Fig. 4B; Table S4). Next, we compared the relative abundance of genotypically antibiotic-resistant isolates for each antibiotic class among *E. faecalis* isolates from hospitalized and nonhospitalized individuals. We found a higher relative abundance of genotypically inferred antibiotic-resistant isolates in hospitalized than in nonhospitalized individuals for aminoglycosides (adjusted *P* = 5.51 × 10^−09^), macrolides (adjusted *P* = 3.77 × 10^−09^), phenicols (adjusted *P* = 8.59 × 10^−05^), and tetracyclines (adjusted *P* = 6.75 × 10^−05^). However, we observed no differences for glycopeptides, i.e., vancomycin (adjusted *P* = 1), which had almost negligible resistance in the isolates (Fig. 4C; Table S4). Again, we observed similar patterns in blood and fecal isolates (Fig. 4D; Table S4).

### There is no evidence of population-wide effects of individual *E. faecalis* genetic changes on infection of individuals by hospitalization status and body isolation source

**Fig 5:**
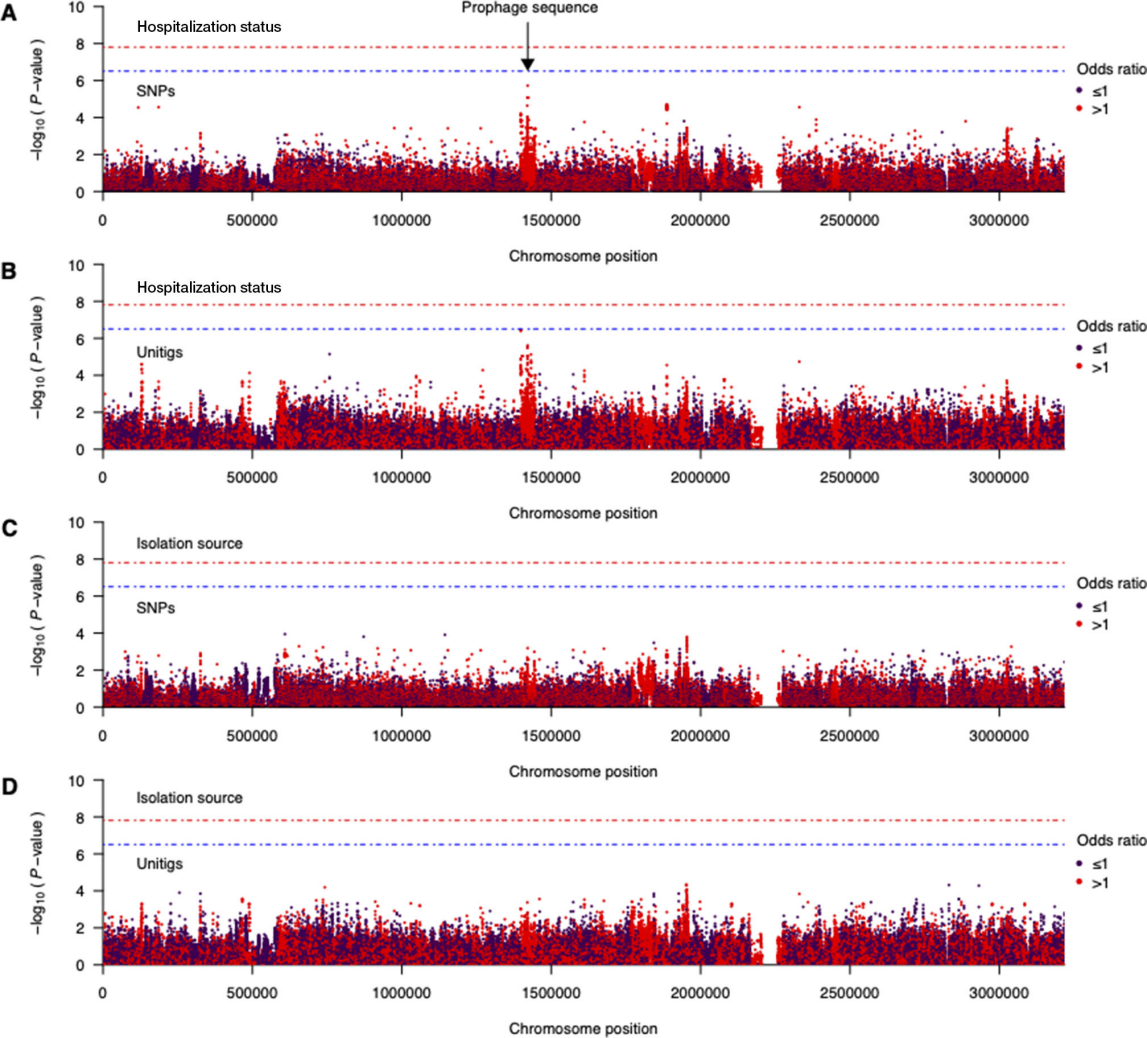
Association of *E. faecalis* genomic variants, hospitalization status, and body isolation source. (**A**) Manhattan plot summarizing the statistical association of SNP with pathogenicity or hospitalization status. The statistical significance of each SNP is log-transformed [−log_10_(*P*-value)] and plotted against its position in the V583 *E. faecalis* reference genome (34 ). (**B**) Manhattan plot summarizing the statistical association of unitigs with pathogenicity. (**C**) Manhattan plot summarizing the statistical association of SNPs with extraintestinal infection or body isolation source. (**D**) Manhattan plot summarizing the statistical association of unitigs with extraintestinal infection or body isolation source. The red and blue dotted lines represent the genome-wide significance and suggestive threshold, respectively.

Having demonstrated differences in the prevalence of virulence factors, likely driven by lineage or strains’ genetic background effects, we next undertook a GWAS using linear mixed models to identify individual *E. faecalis* genetic changes with population-wide events on infection of individuals with varying hospitalization status. We hypothesized that genetic variation in known and unknown virulence factors would be disproportionately distributed among *E. faecalis* isolates from hospitalized and nonhospitalized individuals. In total, we selected 99,730 out of 252,278 SNP variants and 462,374 out of 1,089,909 unitig sequences, which capture variation in both the core and accessory genomes and are present at a frequency of 5% to 95% of the isolates for the GWAS. Contrary to our hypothesis, we found no statistically significant differences in the distribution of SNPs and unitigs between isolates from hospitalized and nonhospitalized individuals independent of the strain genetic background in the GWAS using linear mixed models (50) (Fig. 5A and B). Inspection of the quantile-quantile (Q-Q) plots revealed no issues with population structure (Fig. S1A and B). Altogether, these findings demonstrated that the infection of individuals with varying hospitalization status with *E. faecalis* is not driven by individual genetic changes independently of their genetic background, suggesting that all *E. faecalis* strains are intrinsically adapted for extraintestinal infection, partly through translocation into the bloodstream.

We then carried out an additional GWAS to identify genetic changes associated with extraintestinal infection with *E. faecalis* strains by comparing fecal and bloodstream isolates. Like the GWAS based on the hospitalization status, we found no SNPs and unitigs associated with the isolation source independent of the strains’ genetic background (Fig. 5C and D). However, we found the strongest signal in an ~48.1 Kb genomic region from positions ~1,390,000 to 1,450,000 bp in the V583 *E. faecalis* genome (34 ). Since horizontal gene transfer is a critical process in the mobilization of pathogenicity-associated genes (31, 51), we hypothesized that this region may represent a pathogenicity island. Re-annotation of the nucleotide sequence for this region revealed several phage-associated genes, which suggested the potential integration of a bacteriophage. Similarly, the Q-Q plots showed no issues with adjusting for the population structure (Fig. S1AB). We then performed phage prediction using the entire V583 *E. faecalis* genome sequence to annotate the SNPs and unitig sequences identified in the GWAS. We found a total of nine prophage sequences in the genome, including one with intact *attL*and *attR* attachment sites and integrase sequences located at genomic positions 1,398,051 to 1,446,151 bp. This prophage showed high genetic similarity to prophages including PHAGE_Entero_phiFL3A_NC_013648, PHAGE_Lister_B054_NC_009813 (27), and PHAGE_Lactob_LBR48_NC_027990. Furthermore, most of the phage-associated genes and protein sequences showed high genetic similarity to those found on prophages associated with several bacterial genera, including *Enterococcus*, *Lactobacillus*, *Bacillus*, *Listeria*, and *Staphylococcus*. These findings highlighted a potential virulence locus that should be prioritized for further investigation to understand its role in *E. faecalis* pathogenicity.

## DISCUSSION

Tremendous advances in sequencing technology and analytical approaches have occurred over the past two decades since the sequencing of the first enterococcal genome*—E. faecalis* strain V583 (34). However, despite the increasing availability of population-level *E. faecalis* genomic data sets, no systematic studies have investigated the population-wide effects of individual genetic changes on infection in individuals with varying hospitalization status and extraintestinal infection and the overall contribution of *E. faecalis* genetics to these phenotypes (5). Such studies could reveal critical pathways for *E. faecalis* virulence, including survival in the bloodstream through evasion of innate host immune defenses, and inform the development of therapeutics (12). Here, we address this knowledge gap by investigating the effects of known and novel virulence factors, lineages, and the entire repertoire of *E. faecalis* genomic changes in a large collection of human fecal isolates, representing a snapshot of the *E. faecalis* diversity in the gut, and isolates sampled from blood specimens of individuals with different hospitalization statuses. Our findings demonstrate that the abundance of certain virulence and antibiotic resistance determinants is higher in *E. faecalis* isolates associated with severe disease and extraintestinal infection, largely driven by the effects of the strains, lineages, or genetic background effects but not the population-wide effects of individual genetic changes.

*E. faecalis* is a versatile pathogen that survives in a wide range of challenging niches, including the human gut, blood, and environment, such as in clinical settings. Such adaptation and survival of *E. faecalis* in these diverse environments are modulated by several mechanisms, including antimicrobial resistance (52), intracellular survival (53 – 56), and biofilm formation (27). Although several virulence factors of *E. faecalis* have been described (24 – 27), how (and if this happens) these factors contribute to infection of individuals with varying hospitalization status and extraintestinal infection, especially through gut-to-bloodstream translocation, remains poorly understood. Previous genetic studies shed light on how the distribution of virulence factors shapes the adaptation of *E. faecalis* clones to different environments despite the limitation of small sample sizes (39, 41). In this study, we demonstrate enrichment of known virulence genes in isolates associated with different hospitalization statuses using a larger collection of isolates. These include genes encoding for aggregation substance adherence factors (EF0485 and EF0149) (32); lantipeptide cytolysin subunits CylL-L and CylL-S (*cylL-l* and *cylL-s*), cytolysin subunit modifier (*cylM*), and cytolysin regulator *R2* (*cylR2*) exotoxins (57); and polysaccharide capsule biosynthesis genes (*cpsC* to *cpsK*) involved in immune modulation or antiphagocytosis (58). These findings suggest that the variable abundance of these virulence genes in hospitalized and nonhospitalized individuals could influence *E. faecalis* pathogenicity, possibly because they primarily contribute to intestinal colonization, survival, and fitness or competitiveness in different intestinal compartments in the dysbiotic gut of hospitalized patients. Once the strains harboring these genes are established in higher numbers in the gastrointestinal tract, this promotes transmission, which in turn promotes the evolution and fixation of these virulence genes in the population. Interestingly, the observed higher antibiotic resistance, especially aminoglycosides, in isolates from blood and hospitalized individuals than from feces and nonhospitalized individuals suggests that antibiotic-resistant *E. faecalis* strains are more likely to survive and overgrow after the use of these antibiotics, consistent with findings reported elsewhere (14 – 16, 39, 59, 60). Conversely, while the distribution of the virulence factors and clades, or STs, was observed, the observation from the GWAS of *E. faecalis* pathogenicity, after adjusting for the genetic background of the isolates, implied that no individual genetic changes influence the severity of diseases at the population level. These findings are consistent with the notion that genetic traits influencing virulence are less likely to be selected than those promoting colonization as similarly seen in other pathogens (61). Altogether, these findings suggest that the distribution of the *E. faecalis* virulence factors may largely depend on the genetic background, implying that the lineage effects on pathogenicity may be more pronounced than the population-wide effects of individual genetic changes. Alternatively, there may be a predominance of certain lineages in some individuals, as seen with other opportunistic pathogens (62), whose risk factors for infection, including hospital exposure history, antibiotic treatment, and other underlying conditions, make them favorable for the selection of *E. faecalis* strains enriched in antibiotic resistance genes and other adaptive traits.

Likewise, the distribution of known *E. faecalis* virulence factors by isolation source mirrored the patterns observed for infection in individuals with varying hospitalization status due to the correlation between these phenotypes. These findings suggested that no individual genetic changes are overrepresented in blood and gut niches independent of the genetic background, which implied that while individual genetic changes may have an impact on extraintestinal infection, their effect at the population level is likely minimal. However, some genetic changes could be linked to specific lineages, making disentangling their effects from the genetic background a challenge. However, the absence of genetic changes statistically associated with the body isolation source, after adjusting for the population structure, suggests that these variants are not likely under positive selection because extraintestinal infection represents an evolutionary dead-end for *E. faecalis* (63). Therefore, even if such genetic changes exist, they may be rare and likely exhibit small effect sizes, making their detection challenging without analyzing large data sets with thousands of genomes. We speculate that the observed strong but nonstatistically significant signals in a single prophage, integrated at chromosome coordinates 1,398,051 to 1,446,151 bp in the V583 *E. faecalis* genome (34), could exemplify a potential locus with small population-wide effects on virulence. Indeed, prophages play a critical role in the pathogenicity of *E. faecalis* (64 – 67) and other bacterial pathogens, such as *Staphylococcus aureus* (37, 68). Therefore, further studies using even larger genomic data sets than the present study and adjusting for other important covariates, such as prior antibiotic usage and immune status, are required to fully investigate the impact of the identified *E. faecalis* prophage in modulating extraintestinal infection. Crucially, such studies should prospectively collect samples to minimize confounding effects due to cohort and temporal variability between the number of cases and controls for a robust GWAS, which was one of the limitations of this study. Furthermore, definitive *E. faecalis* genetic signals for extraintestinal infection may be identified by comparing isolates obtained from the blood of patients with feces from individuals with confirmed negative blood cultures as controls. Inclusion of *E. faecalis* strains from community-acquired infections could also overcome the confounding effects due to factors related to hospitalization, such as *E. faecalis* from individuals with community-acquired bacteremia who are at a higher risk of developing infective endocarditis (69). Altogether, our findings demonstrate that no individual *E. faecalis* genetic changes exhibit a population-wide statistical association with extraintestinal infection, implying that all *E. faecalis* strains are capable of translocating into the bloodstream and causing severe diseases, consistent with their known opportunistic pathogenic lifestyle. Although *E. faecalis* genetic changes that are important for survival in the blood may exist, these would not be fixed in the population, especially if they had no impact on colonization, as individual strains would have to accidentally “re-discover” them repeatedly. Therefore, vaccination strategies targeting all rather than specific genetic backgrounds would lead to increased protection from severe *E. faecalis* diseases.

The estimated heritability based on unitig sequence variation of ~40% for infection in individuals with different hospitalization statuses and ~30% for body isolation source suggests that the contribution of *E. faecalis* genetics to these phenotypes is not negligible but relatively modest compared to that observed for other phenotypes, such as antimicrobial resistance (70). Our findings are consistent with findings from a recent bacterial GWAS of pathogenicity in *Streptococcus pneumoniae* (71) and Group B Streptococcus (*Streptococcus agalactiae*) (72). However, other studies have found negligible heritability for pathogenicity in *Neisseria meningitidis* (61), which suggests that the evolution of the pathogenicity trait is neutral. Previous studies have suggested that antibiotic resistance plays a major role in bloodstream invasion (14 – 16, 59, 60). Indeed, broad-spectrum antibiotic use disrupts the stable gut microbial community by removing typically antibiotic-susceptible competitor species, leading to the overgrowth and dissemination of *E. faecalis* into the bloodstream (59, 60). Therefore, follow-up studies of *E. faecalis* isolates sampled from feces of healthy individuals and bloodstream of patients, adjusting for other important variables such as antibiotic use, are required to determine specific genetic changes modulating pathogenicity and virulence and account for potential missing heritability. These studies will be better placed to assess the relative effect of host and gut environmental factors, such as microbiota perturbations due to antibiotic use, compared to the population-wide impact of individual genetic changes in modulating *E. faecalis* virulence and pathogenicity (73).

We acknowledge the limitations of this study, which primarily stem from the sampling biases due to the use of preexisting sequencing data sets. Firstly, there was uneven distribution of blood and fecal isolates from hospitalized and nonhospitalized individuals. Secondly, due to the retrospective nature of the study, we did not have access to detailed clinical information, including comorbidities, previous antibiotic use, and the individual’s age. Adjusting for these factors would further strengthen our findings. Thirdly, our sample size is modest as it is based on a collection of *E. faecalis* isolates from only two countries in Europe. However, our data set size is similar to or larger than those described in previous studies (68, 74), which demonstrated sufficient power to detect statistically significant associations between specific individual loci and phenotypes. We recommend follow-up studies with larger sample sizes, balanced data sets by hospitalization status and body isolation source, and most importantly, including detailed clinical information, especially antibiotic use, comorbidities, and an individual’s age, to adjust for potential confounding effects in the GWAS analysis.

Our exploratory findings derived from a geographically and temporally diverse whole-genome data set of *E. faecalis* isolates suggest that the pathogenicity of *E. faecalis* infections may not be primarily driven by the specific population-wide effects of individual genetic changes. These results may further illustrate the opportunistic pathogenic lifestyle of *E. faecalis*, whereby infection of individuals with different hospitalization statuses and body isolation sources could be an accidental consequence of gut colonization dynamics as seen in other gut commensals (63). Due to the absence of specific individual genetic variants associated with body isolation source and hospitalization status, ultimately, the commensal-to-pathogen switch and virulence of *E. faecalis* may be predominantly modulated by multiple genetic variants, i.e., polygenic, genetic background or lineages, epigenetic mechanisms, host factors, and the gut milieu, including the ecological side effects of broad-spectrum antibiotics on the gastrointestinal microbiota.

## MATERIALS AND METHODS

### Sample characteristics and microbiological processing

For this study, we selected a total of 736 human *E. faecalis* isolates from a collection of whole-genome sequences from isolates collected from several European countries described by Pöntinen et al. (43). We included isolates from countries where both fecal and blood specimens were collected, namely, the Netherlands (*n* = 300) and Spain (*n* = 436). The isolates represent collections from the University Medical Center Utrecht, Utrecht, The Netherlands (*n* = 300); the European Network for Antibiotic Resistance and Epidemiology at the University Medical Center Utrecht, Utrecht, The Netherlands (*n* = 6); the Hospital Ramòn y Cajal, Madrid, Spain (*n* = 375); and Spain (*n* = 55). By isolation source, 296 isolates were sampled from feces, while 440 were from blood. Of these, 485 were collected from hospitalized patients, while 251 were from nonhospitalized individuals. The isolates were collected over a 21-year period (1996 to 2016); therefore, our data set was both geographically and temporally diverse. We did not use clinical metadata related to the patients, and all isolate identifiers were de-identified; therefore, additional institutional review board approval was not required.

### Genome sequencing, molecular typing, assembly, and annotation

Short-read sequencing was done at the Wellcome Sanger Institute using the Illumina HiSeq X paired-end sequencing platform. As part of our quality control procedures, we used Kraken (version 0.10.66) (75) to check for potential species contamination. We assembled sequence reads that passed quality control using Velvet *de novo* assembler (version 1.2.10) (76) and annotated the resultant draft assemblies using Prokka (version 1.14.6) (77). To generate multiple sequence alignments for the whole genome sequences, we mapped the reads against the V583 *E. faecalis* reference genome (34) using the Snippy (version 4.6.0) haploid variant calling and core genome pipeline (https://github.com/tseemann/snippy). We performed *in silico* genome-based typing of the isolates using MLST, using ST or clone definitions in the MLST database (https://pubmlst.org/efaecalis) ( 48, 78), implemented in SRST2 (79).

### Phylogenetic reconstruction and population structure analysis

To generate a phylogeny of the *E. faecalis* isolates, we first identified genomic positions containing SNPs using SNP-sites (version 2.3.2) (80). Next, we used the SNPs to construct a maximum-likelihood phylogenetic tree using IQ-TREE (version 2.1.2) (81). We selected the general time reversible and Gamma substitution models. We processed and rooted the generated phylogeny at the midpoint of the longest branch using the APE package (version 4.3) (82) and phytools (version 0.7.70) (83). We annotated and visualized the rooted phylogeny using the “gridplot” and “phylo4d” functions implemented in phylosignal (version 1.3) (84) and phylobase (version 0.8.6) packages (https://cran.r-project.org/package=phylobase), respectively. We used PopPUNK (version 1.2.2) to define the population structure of the isolates (47).

### Antibiotic resistance and virulence gene profiles

We identified genotypic antibiotic resistance for seven major antibiotic classes, namely, glycopeptides (vancomycin), aminoglycosides, macrolides, tetracyclines, phenicols, and oxazolidinones, as described by Pöntinen et al. (43). We screened the sequencing reads for the presence and absence of antibiotic resistance genes using ARIBA (version 2.14.4) (85) and the ResFinder 3.2 database (86). We included additional genes conferring resistance to vancomycin, namely, vanA [European Nucleotide Archive (ENA): accession: AAA65956.1], vanB (ENA accession: AAO82021.1), vanC (ENA accession number: AAA24786.1), vanD (ENA accession: AAD42184.1), vanE (ENA accession: AAL27442.1), and vanG (ENA accession: NG_048369.1), and linezolid, namely, cfrD (ENA accession: PHLC01000011). We compared the abundance of antibiotic resistance genes per isolate using a generalized linear regression model with a Poisson log link function with pathogenicity or hospitalization status and country of origin as covariates, the latter to adjust for geographical differences. We used the test of equal proportions to compare the relative abundance of genotypic antibiotic resistance for each antibiotic class among hospitalized and nonhospitalized individuals, as well as blood and feces.

We also assessed the presence and absence of *E. faecalis* virulence genes obtained from the VFDB (49). These included genes encoding proteins involved in adherence to the epithelial surfaces (*ace*, *ebpA*, *ebpB*, *ebpC*, *ecbA*, EF0149, EF0485, *efaA*, and *srtC*), exoenzymes (EF0818, EF3023, *gelE*, and *sprE*), biofilm formations (*bopD*, *fsrA*, *fsrB*, and *fsrC*), immune modulation or antiphagocytosis (*cpsA-K*), and exotoxins (*cylL-l*, *cylL-s*, *cylM*, and *cylR2*) between isolates from hospitalized and nonhospitalized individuals and those associated with intestinal colonization and extraintestinal infection. We used BLASTN (version 2.9.0+) (87) to determine the presence and absence of the virulence genes. To avoid incorrectly missing genes potentially split between multiple contigs during *de novo* genome assembly, we considered all the highest scoring pairs with a minimum length of 100 bp using BioPython (88). We used the test of equal proportions to compare the relative abundance of genotypic antibiotic resistance for each antibiotic class among hospitalized and nonhospitalized individuals, as well as blood and feces.

### Genome-wide association study

To generate the input SNP data for the GWAS, we used VCFtools (version 0.1.16) (89) to convert bi-allelic SNPs into the pedigree file accepted by PLINK software (90). We filtered out genomic positions with SNPs with a minor allele frequency of <5% or missing variant calls in >10% of the isolates using PLINK (version 1.90b4) (90). Next, we identified unitig sequences, variable-length *k-*mer sequences generated from nonbranching paths in a compacted De Bruijn graph. First, we build a De Bruijn graph using assemblies of all the isolates based on 31 bp *k*-mer sequences using Bifrost (version 1.0.1) (91). We then queried the generated De Bruijn graph using the query option in Bifrost to generate the presence and absence patterns of each identified unitig in the assemblies of each isolate. We then combined the presence and absence patterns of all the isolates into a single file and then merged them with the phenotype data (isolation source or hospitalization status) to generate PLINK-formatted pedigree files, which were used for the downstream GWAS analysis. We used the same threshold for variant frequency to filter out rare unitigs before the GWAS.

We undertook GWAS analyses using SNPs and unitigs to identify genetic variants associated with pathogenicity (hospitalization) and extraintestinal infection of *E. faecalis.* We used FaST-LMM (FastLmmC, version 2.07.20140723) (50), which uses a linear mixed model for the GWAS. For both methods, we specified a kinship matrix based on the unitig presence and absence data to adjust for the clonal population structure of the isolates, which is a major confounder in bacterial GWAS analyses (35). Since the GWAS tools used in this study were originally developed to mostly handle human diploid DNA data, we coded the variants as human mitochondrial DNA (which is haploid) by specifying the chromosome number as 26 (92, 93). To control the false discovery rate, we used the Bonferroni correction method to adjust the statistical significance (*P*-values) inferred by each GWAS method based on the likelihood ratio test. We specified the genome length of the *E. faecalis* V583 reference genome (3,218,031 bp) as the maximum possible number of genomic variants possible, assuming that variants can independently occur at each genomic position. Since this assumption may not necessarily be true, our approach is likely to be more conservative than the Bonferroni correction based on the number of tested variants; therefore, it may minimize false positives but may slightly increase false negatives. The advantage of our approach is that by using the same number of possible variants based on the genome length, a consistent *P*-value threshold can be used to adjust different types of genetic variation, i.e., SNPs, accessory genes, *k-*mers, and unitigs, to simplify interpretation and comparison of statistical significance across different studies.

We visualized the GWAS results using Manhattan plots generated using standard plotting functions in R (version 4.0.3) (https://www.R-project.org/). Specific genomic features associated with each SNP and unitig were analyzed further by comparing the genomic sequences to the V583 *E. faecalis* reference genome (34) using BLASTN (version 2.5.0+) (94) and BioPython (version 1.78) (88). To identify potential issues arising due to the population structure, we generated Q-Q plots to compare the observed and expected statistical significance using qqman (version 0.1.7) (95). We calculated the overall proportion of the variance of the phenotype explained by *E. faecalis* genetics, i.e., narrow-sense heritability, using GCTA (version 1.93.2) (44).

### Statistical analysis

We compared the number of *E. faecalis* antibiotic resistance genes per isolate among hospitalized and nonhospitalized patients and blood and fecal isolates using a Poisson generalized linear regression model with a log link. We used the rest for equality of proportions to assess whether a single dominant lineage is present at a frequency of >50%. We compared the frequency of STs and lineages in isolates from hospitalized and nonhospitalized patients and blood and fecal isolates using the chi-squared test.

## Data Availability

The whole-genome sequencing data used in this study are publicly available in the European Nucleotide Archive (ENA) under the accession numbers provided in Data Set S1 in the supplemental material.

## SUPPLEMENTAL MATERIAL

Data Set S1 spectrum.00201-23-s0001.xlsx Characteristics of the whole-genome sequenced *E. faecalis* isolates used in the present study.

Supplemental material spectrum.00201-23-s0002.pdf Tables S1 to S4 and Fig. S1.

ASM does not own the copyrights to Supplemental Material that may be linked to, or accessed through, an article. The authors have granted ASM a non-exclusive, world-wide license to publish the Supplemental Material files. Please contact the corresponding author directly for reuse.
