# Supplementary material for: The population-level impact of Enterococcus faecalis genetics on intestinal colonization and extraintestinal infection
Source: Microbiol Spectr. 2023 Oct 9;11(6):e00201-23. doi: 10.1128/spectrum.00201-23 (PMC10714801; doi:10.1128/spectrum.00201-23)
Supplement: Supplemental material — Tables S1 to S4 and Fig. S1. [file spectrum.00201-23-s0002.pdf]

The population-level impact of *Enterococcus faecalis* genetics on intestinal colonisation and extraintestinal infection

Chrispin Chaguza, Anna K. Pöntinen, Janetta Top, Sergio Arredondo-Alonso, Ana R. Freitas, Carla Novais, Carmen Torres, Stephen D. Bentley, Luisa Peixe, Teresa M. Coque, Rob J.L. Willems, and Jukka Corander

Supplementary Table 1. Relative abundance of *E. faecalis* clades or lineages by individuals' hospitalisation status and isolation source.

PopPUNK clade	Proportion by isolation source		Bonferroni adjusted *P*-value	Proportion by hospitalisation status		Bonferroni adjusted *P*-value
	Blood	Faeces		Hospitalised	Non-hospitalised	
1	0.11 (55/499)	0.17 (43/251)	0.026	0.1 (45/452)	0.18 (53/298)	0.0027
11	0.016 (8/499)	0.008 (2/251)	0.57	0.015 (7/452)	0.01 (3/298)	0.76
13	0.036 (18/499)	0.024 (6/251)	0.5	0.035 (16/452)	0.027 (8/298)	0.66
15	0.02 (10/499)	0.012 (3/251)	0.61	0.02 (9/452)	0.013 (4/298)	0.7
16	0.012 (6/499)	0.024 (6/251)	0.36	0.011 (5/452)	0.023 (7/298)	0.3
17	0.02 (10/499)	0.032 (8/251)	0.46	0.022 (10/452)	0.027 (8/298)	0.87
2	0.16 (80/499)	0.024 (6/251)	6.30E-08	0.16 (71/452)	0.05 (15/298)	1.20E-05
20	0.02 (10/499)	0 (0/251)	0.055	0.022 (10/452)	0 (0/298)	0.024
22	0.01 (5/499)	0.02 (5/251)	0.44	0.011 (5/452)	0.017 (5/298)	0.73
3	0.082 (41/499)	0.06 (15/251)	0.34	0.088 (40/452)	0.054 (16/298)	0.1
4	0.034 (17/499)	0.19 (48/251)	1.40E-12	0.033 (15/452)	0.17 (50/298)	3.40E-10
5	0.038 (19/499)	0.048 (12/251)	0.66	0.04 (18/452)	0.044 (13/298)	0.95
6	0.12 (58/499)	0 (0/251)	4.30E-08	0.12 (55/452)	0.01 (3/298)	4.80E-08
7	0.056 (28/499)	0.008 (2/251)	0.0029	0.062 (28/452)	0.0067 (2/298)	0.00033
8	0.03 (15/499)	0.036 (9/251)	0.84	0.029 (13/452)	0.037 (11/298)	0.68
9	0.026 (13/499)	0.028 (7/251)	1	0.02 (9/452)	0.037 (11/298)	0.24

Supplementary Table 2. Relative abundance of *E. faecalis* sequence types (ST) and virulence factors by individuals' hospitalisation status and isolation source.

Sequence type (ST)	Proportion by isolation source		Bonferroni adjusted *P*-value	Proportion by hospitalisation status		Bonferroni adjusted *P*-value
	Blood	Faeces		Hospitalised	Non-hospitalised	
ST16	0.076 (38/499)	0.06 (15/251)	0.5	0.082 (37/452)	0.054 (16/298)	0.18
ST179	0.026 (13/499)	0.028 (7/251)	1	0.02 (9/452)	0.037 (11/298)	0.24
ST21	0.038 (19/499)	0.06 (15/251)	0.25	0.038 (17/452)	0.057 (17/298)	0.28
ST25	0.012 (6/499)	0.068 (17/251)	7.80E-05	0.013 (6/452)	0.057 (17/298)	0.0014
ST28	0.11 (53/499)	0 (0/251)	1.90E-07	0.12 (52/452)	0.0034 (1/298)	1.20E-08
ST34	0.028 (14/499)	0.02 (5/251)	0.67	0.029 (13/452)	0.02 (6/298)	0.62
ST40	0.044 (22/499)	0.092 (23/251)	0.015	0.04 (18/452)	0.091 (27/298)	0.0068
ST55	0.038 (19/499)	0.024 (6/251)	0.42	0.033 (15/452)	0.034 (10/298)	1
ST6	0.15 (76/499)	0.024 (6/251)	2.10E-07	0.15 (68/452)	0.047 (14/298)	1.50E-05
ST64	0.024 (12/499)	0.024 (6/251)	1	0.024 (11/452)	0.023 (7/298)	1
ST9	0.038 (19/499)	0.008 (2/251)	0.034	0.042 (19/452)	0.0067 (2/298)	0.0082

Supplementary Table 3. Relative abundance of *E. faecalis* virulence factors by individuals' hospitalisation status and isolation source.

Gene	Type	Proportion by isolation source		Bonferroni adjusted *P*-value	Proportion by hospitalisation status		Bonferroni adjusted *P*-value
		Blood	Faeces		Hospitalised	Non-hospitalised	
ace	Adherence	1.00 (439/440)	1.00 (296/296)	1	1.00 (484/485)	1.00 (251/251)	1
ebpA	Adherence	0.99 (437/440)	1.00 (296/296)	0.4	0.99 (482/485)	1.00 (251/251)	0.52
ebpB	Adherence	1.00 (438/440)	1.00 (296/296)	0.66	1.00 (483/485)	1.00 (251/251)	0.79
ebpC	Adherence	1.00 (438/440)	1.00 (296/296)	0.66	1.00 (483/485)	1.00 (251/251)	0.79
ecbA_fss3	Adherence	0.55 (240/440)	0.38 (113/296)	1.80E-05	0.55 (268/485)	0.34 (85/251)	5.70E-08
EF0149	Adherence	0.68 (297/440)	0.55 (163/296)	0.00084	0.67 (323/485)	0.55 (137/251)	0.0019
EF0485	Adherence	0.68 (297/440)	0.55 (164/296)	0.0012	0.67 (323/485)	0.55 (138/251)	0.0026
efaA	Adherence	1.00 (440/440)	1.00 (296/296)	1	1.00 (485/485)	1.00 (251/251)	1
srtC	Adherence	1.00 (438/440)	1.00 (296/296)	0.66	1.00 (483/485)	1.00 (251/251)	0.79
bopD	Biofilm	1.00 (440/440)	1.00 (296/296)	1	1.00 (485/485)	1.00 (251/251)	1
fsrA	Biofilm	0.57 (249/440)	0.51 (152/296)	0.19	0.57 (276/485)	0.50 (125/251)	0.079
fsrB	Biofilm	0.58 (254/440)	0.52 (154/296)	0.15	0.58 (281/485)	0.51 (127/251)	0.069
fsrC	Biofilm	0.86 (378/440)	0.77 (229/296)	0.0038	0.86 (416/485)	0.76 (191/251)	0.0015

EF0818	Exoenzyme	0.68 (297/440)	0.61 (180/296)	0.074	0.68 (331/485)	0.58 (146/251)	0.0085
EF3023	Exoenzyme	0.79 (349/440)	0.86 (255/296)	0.023	0.79 (385/485)	0.87 (219/251)	0.011
gelE	Exoenzyme	0.86 (379/440)	0.78 (232/296)	0.0081	0.86 (418/485)	0.77 (193/251)	0.0021
sprE	Exoenzyme	0.88 (388/440)	0.81 (240/296)	0.01	0.88 (428/485)	0.80 (200/251)	0.0027
cylL-l	Exotoxin	0.37 (163/440)	0.23 (67/296)	5.00E-05	0.37 (181/485)	0.20 (49/251)	1.20E-06
cylL-s	Exotoxin	0.37 (163/440)	0.23 (67/296)	5.00E-05	0.37 (181/485)	0.20 (49/251)	1.20E-06
cylM	Exotoxin	0.37 (163/440)	0.23 (67/296)	5.00E-05	0.37 (181/485)	0.20 (49/251)	1.20E-06
cylR2	Exotoxin	0.37 (163/440)	0.23 (67/296)	5.00E-05	0.37 (181/485)	0.20 (49/251)	1.20E-06
cpsA	Immune modulation	1.00 (440/440)	1.00 (296/296)	1	1.00 (485/485)	1.00 (251/251)	1
cpsB	Immune modulation	1.00 (440/440)	1.00 (296/296)	1	1.00 (485/485)	1.00 (251/251)	1
cpsC	Immune modulation	0.63 (276/440)	0.41 (120/296)	5.10E-09	0.61 (298/485)	0.39 (98/251)	1.20E-08
cpsD	Immune modulation	0.63 (276/440)	0.40 (119/296)	3.00E-09	0.61 (298/485)	0.39 (97/251)	6.60E-09
cpsE	Immune modulation	0.63 (276/440)	0.40 (119/296)	3.00E-09	0.61 (298/485)	0.39 (97/251)	6.60E-09
cpsF	Immune modulation	0.44 (194/440)	0.27 (79/296)	2.40E-06	0.44 (211/485)	0.25 (62/251)	8.40E-07
cpsG	Immune modulation	0.63 (276/440)	0.40 (119/296)	3.00E-09	0.61 (298/485)	0.39 (97/251)	6.60E-09

cpsH	Immune modulation	0.63 (276/440)	0.40 (119/296)	3.00E-09	0.61 (298/485)	0.39 (97/251)	6.60E-09
cpsI	Immune modulation	0.63 (276/440)	0.41 (120/296)	5.10E-09	0.61 (298/485)	0.39 (98/251)	1.20E-08
cpsJ	Immune modulation	0.63 (276/440)	0.41 (120/296)	5.10E-09	0.61 (298/485)	0.39 (98/251)	1.20E-08
cpsK	Immune modulation	0.63 (276/440)	0.41 (120/296)	5.10E-09	0.61 (298/485)	0.39 (98/251)	1.20E-08

Supplementary Table 4. The abundance of *E. faecalis* antibiotic resistance genes by individuals' hospitalisation status and isolation source.

Antibiotic	Body isolation source		Bonferroni adjusted P-value	Hospitalisation status		Bonferroni adjusted P-value
	Blood	Faeces		Hospitalised	Non-hospitalised	
Aminoglycoside	0.51 (225/440)	0.28 (84/296)	5.51E-09	0.51 (247/485)	0.25 (62/251)	5.69E-11
Linezolid	0 (0/440)	0 (0/296)	1	0 (0/485)	0 (0/251)	1
Macrolide	0.53 (233/440)	0.3 (88/296)	3.77E-09	0.53 (256/485)	0.26 (65/251)	2.70E-11
Phenicol	0.29 (127/440)	0.16 (46/296)	8.59E-05	0.28 (136/485)	0.15 (37/251)	0.00016145
Tetracycline	0.75 (328/440)	0.59 (176/296)	6.75E-05	0.75 (364/485)	0.56 (140/251)	4.52E-07
Vancomycin	0.0091 (4/440)	0.027 (8/296)	0.11246715	0.016 (8/485)	0.016 (4/251)	1

*All the values in the contingency table were increased by a value of 1 to avoid division by zero when calculating the *P*-values.

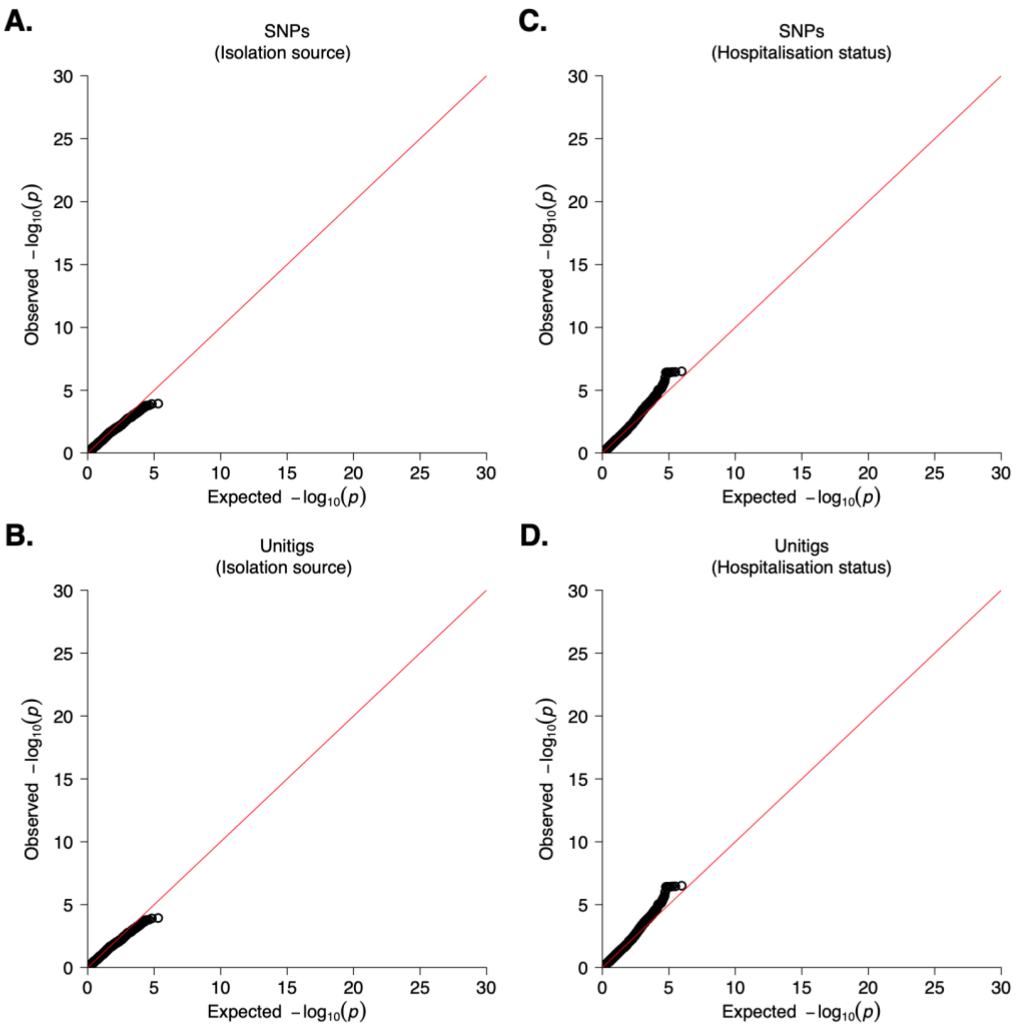

Supplementary Figure 1. Quantile-quantile plots for the genome-wide association (GWAS) of *E. faecalis* body isolation site and hospitalisation status. **(A)** QQ-plot of GWAS based on SNPs for the isolation source phenotype. **(B)** QQ-plot of GWAS based on unitigs for the isolation source phenotype. **(C)** QQ-plot of GWAS based on SNPs for the hospitalisation status phenotype. **(D)** QQ-plot of GWAS based on unitigs for the hospitalisation status phenotype.
